# Opioids: an unexplored option for treatment of dyspnea in IPF

**DOI:** 10.3402/ecrj.v3.30629

**Published:** 2016-03-10

**Authors:** Charlotte Kohberg, Charlotte Uggerhøj Andersen, Elisabeth Bendstrup

**Affiliations:** 1 Department of Respiratory Diseases and Allergy, Aarhus University Hospital, Aarhus, Denmark; 2 Department of Clinical Pharmacology, Aarhus University Hospital, Aarhus, Denmark

**Keywords:** interstitial lung disease, chronic obstructive lung disease, COPD, morphine, opioid, dyspnea, review

## Abstract

**Background:**

Idiopathic pulmonary fibrosis (IPF) is the most common among the idiopathic interstitial pneumonias and has the worst prognosis, with a median survival of 3–5 years. The most common symptom in IPF is dyspnea, impacting on the patient's quality of life and life expectancy. Morphine in the treatment of dyspnea has been investigated but with conflicting results. This review aims to clarify the role of opioids in the treatment of dyspnea in patients with IPF.

**Methods:**

A literature search was performed using the MeSH and PubMed databases. As only very few studies included patients with IPF, studies conducted primarily with patients with chronic obstructive pulmonary disease were also included. In total, 14 articles were found.

**Results:**

Seven studies reported use of systemic morphine and seven studies of inhaled morphine. Five of the seven studies investigating systemic administration detected an improvement in either dyspnea or exercise capacity, whereas no beneficial effect on dyspnea was detected in any study using inhaled morphine. No severe adverse effects such as respiratory depression were reported in any study, although constipation was reported as a notable adverse effect.

**Conclusions:**

Results were inconsistent, but in some studies systemic morphine administration showed a significant improvement in the dyspnea score on a visual analog scale without observation of severe side effects. Nebulized morphine had no effect on dyspnea.

In interstitial idiopathic pneumonia, idiopathic pulmonary fibrosis (IPF) is the most common, with fewer than 150 cases annually in Denmark (1). IPF has the lowest survival rate among the interstitial lung diseases (ILDs) (2) with a median survival of 3–5 years. There is no curative treatment, but early diagnosis is important to slow down disease progression with anti-fibrotic medication (3). The most prominent symptom of IPF is dyspnea, although coughing, fatigue, anxiety, depression, chest pain, and weight loss also affect patients with IPF (4). This review focuses on dyspnea due to the huge impact it has on the quality of life, functional level, social relationships, feeling of independence, overall well-being (5), and prognosis of patients with IPF (6, 7).

The American Thoracic Society defines dyspnea as ‘a subjective experience of breathing discomfort that consists of qualitatively distinct sensations that vary in intensity’ (8). The mechanism behind dyspnea has not yet been fully clarified but is thought to be caused by a combination of peripheral as well as central nervous mechanisms. The sensory afferent sources available for respiratory sensation are multiple. Not only peripheral sources of input but also corollary discharge from respiratory areas in the brain contribute information on respiration to perceptual areas. Corollary discharge arises from the brain stem and cortical sources, controlling both automatic and involuntary responses to sensory input and delivering a descending as well as an ascending response. It has been proved that different combinations of afferent inputs lead to different sensations (9).

Neuroimaging by MRI has visualized how dyspnea and pain activate common areas in the brain such as the bilateral anterior and mid-insula, left amygdala, and right medial thalamus (10). Drugs used for treatment of pain are known to target some of these areas and it is reasonable to believe that therapy directed to these common regions could inhibit not just the sensation of pain but also the sensation of dyspnea. Furthermore, studies have demonstrated participation of the insula, anterior cingulate cortex, and amygdala in the processing of emotions such as anxiety and fear, indicating that dyspnea and pain share a cerebral network. This overlap supports the observation of patients managing dyspnea or pain differently depending on their level of anxiety or fear, and it incites the management of the emotional as well as symptomatic aspects. The opioid morphine is one of the commonly used drugs with a centrally acting pain-relieving effect. Morphine might be used to inhibit the central perception of dyspnea. Furthermore, a simultaneous local effect of morphine may be possible as opioid receptors have been detected in the respiratory tract (11). A laboratory test with blockade of endogenous opioids with naloxone in patients with chronic obstructive pulmonary disease (COPD) during exercise on a treadmill supports the thesis of a central effect (12). Naloxone had no effect on exercise tolerance, and the respiratory response was without change in VO_2_ and VE compared with saline treatment, but still the central perception of shortness of breath was worse. Thus, theoretically it is possible that morphine could act locally in the respiratory tract to lower the level of sensory input to the perceptual areas; act centrally to lower emotions of anxiety and fear, thereby lowering the symptoms of dyspnea; and finally directly affect the central perceptual processing and thus the total sensation of dyspnea.

This review investigates the current knowledge of the use of opioids in the treatment of dyspnea in patients with IPF. The data on opioid treatment for dyspnea in IPF is limited (13–15). As a consequence of this, the literature search was broadened from initially only including studies with IPF patients to also include studies of other chronic lung diseases.

## Methods

A literature search was performed in October 2015 in the PubMed and MeSH databases. A search builder was created using a combination of the following terms: *IPF*, *dyspnea*, *treatment*, *pulmonary fibrosis*, *lung fibrosis*, *obstructive pulmonary disease*, *morphine*, and *opioid*. Consequently, filters were used to include only human study groups and English-language articles. As this search resulted in very few articles, the MeSH term *obstructive pulmonary disease* was included. The mechanism and expression of dyspnea as well as the response to opioid treatment in patients with COPD were considered to be the most comparable with patients with IPF (15), although differences have been detected as well (16). Studies including patients with cancer and heart disease were excluded.

Sorted by title and abstract, 38 articles were found, including 14 reviews. The reviews were used as background theory. After rejecting duplicates and non-relevant articles, 14 original articles were included in this review (Table 1).

**Table 1:** Characteristics of studies investigating the effect of morphine on dyspnea

Reference	Study type	Study group	Drug, dose, and route of administration	Equivalent oral morphine dose	Measures: primary outcome (secondary outcome)	Results
Johnson et al., British Med. J. 1983 (26)	Randomized, double-blinded, placebo-controlled crossover study	17 COPD patients and one with pulmonary fibrosis.	Oral 15 mg dihydrocodeine t.i.d. for 1 week and on alternate days for 1 week (i.e. treatment for 2 weeks).	3 mg	100 mm VAS score for dyspnea; daily and during treadmill test (PEFR, number of steps, VAS score for breathlessness, constipation, nausea, anxiety, and drowsiness at home. FEV1, FVC, and distance and VAS score for dyspnea at treadmill).	Reduced dyspnea both weekly and on alternate days (*p*<0.001). Longer walk distance after 1 week of treatment (*p*<0.05). Reduced dyspnea at treadmill test.
Eiser et al., Eur Respir J 1991 (27) First study^a^	Randomized, double-blinded, placebo-controlled crossover study	18 COPD patients.	Oral linctus of diamorphine 2.5 mg or 5 mg as 5 ml for 2 weeks each.	10 or 20 mg	100 mm VAS scale for dyspnea; daily and at 6MWT and treadmill walk on study day (VAS scale for drowsiness, overall well-being, and number of times awakening during the night because of dyspnea. Spirometry, blood gases, plasma level of morphine. Walk distance at 6MWT, time on treadmill.)	No significant difference in dyspnea at home or related to exercise tests.
Second study^a^	Randomized, double-blinded, placebo-controlled, crossover study	10 COPD patients.	Oral linctus of diamorphine 7.5 mg.	30 mg	100 mm VAS scale for dyspnea at 6MWT (spirometry, blood gases, plasma level of morphine, distance at 6MWT).	No statistical significance in changes, but trend for both dyspnea and walking distance to improve on morphine.
Light et al., Chest 1996 (23) First study^a^	Randomized, double-blinded, placebo-controlled crossover study	Seven COPD patients.	Oral solution of morphine 30 mg alone, 30 mg morphine and 25 mg MP or 30 mg morphine and 10 mg MPC.	30 mg	Modified Borg score for dyspnea during the last 30 sec of every power output at the cycle ergometer test. (Subjective feelings with Bond scale, visual vigilance, motor speed and spirometry. Expired gas analysis, HR, and RR.)	Improved dyspnea with MP treatment (except at VO_2_max), not with M alone. Significant increase in VO_2_max and decrease in VO_2_ and VCO_2_ after MP.
Second study	Randomized, double-blinded, placebo-controlled crossover study	Nine COPD patients.	Oral solution of 25 mg promethazine (P) or 30 mg morphine and 25 mg promethazine (MP).	30 mg		No significant difference in dyspnea. Significant increase in VO_2_max, VCO_2_max, VE, and VT.
Poole et al., Am. J. Respir. Crit. Care Med. 1998 (19)	Randomized, double-blinded, placebo-controlled, crossover study	16 COPD patients.	Oral sustained-release morphine sulfate 10 mg tablets – titrated from 10 mg once a day to maximally 20 mg twice a day for 6 weeks.	20 mg×2	Chronic Respiratory Disease Questionnaire (QRQ) subscale for dyspnea at rest both at home twice daily and at study center in the beginning and end of each 6-week treatment period (FEV1, FVC, distance at 6MWT, sat − O_2_, and side effects).	No significant improvement in dyspnea. Distance at 6MWT decreased during morphine treatment, but increased during placebo.
Abernethy et al., British Med. J 2003 (21)	Randomized, double-blinded, placebo-controlled crossover study	38 COPD patients, 10 not specified.	Oral sustained-release morphine sulfate q.d. 20 mg for 4 days.	5 mg	100 mm VAS scale for dyspnea in the evening at rest (morning dyspnea, exercise tolerance, RR, BP, HR, sat-O_2_, disturbance of sleep. Categorical scale for 7 items.)	Reduced dyspnea in the morning (*p*=0.011) and evening (*p*=0.006). Less sleep disturbance (*p*=0.039).
Currow et al., J. of Pain and Symptom Management 2011 (17)	Open-label, Phase II dose increment study followed by Phase IV effectiveness and safety study	83 outpatients (54% with COPD, 12% with ILD, the rest with different diagnoses). 52 joined the Phase IV study.	Oral morphine: initially 10 mg daily raised to maximally 30 mg daily for non-responders for 3 months if no side effects.	10–30 mg	100 mm VAS scale for dyspnea twice daily at rest (quality of life, number of dose changes, dose at 3 months, side effects).	Reduced dyspnea in both phases. Benefit maintained for >3 months for one-third of patients. Decreased VAS score with 17.1 mm (SD 11.6).
Allen et al., Pall. Med. 2005 (14)	Non-randomized, open case study	11 IPF patients.	Subcutaneously injected q.d. 2.5/5 mg diamorphine (5 mg for patients >60 kg). Oral morphine as long-term treatment. Mean survival of 5 weeks after starting opioids.	10 or 20 mg	100 mm VAS score for dyspnea at rest (HR, BP, RF, sat-O_2_).	Reduced dyspnea (*p*<0.0001).
Young et al., Thorax 1989 (18)	Randomized, double-blind, placebo-controlled crossover study	11 patients (2 IPF, 9 COPD).	Nebulized 5 mg of morphine (1 g/ml).	5 mg	No dyspnea score used. Dyspnea only recorded as a limiting symptom at cycle ergometer test (spirometry).	Dyspnea maintained to be the limiting symptom in all three tests. Mean endurance time increased with morphine.
Harris-Eze et al., Am. J. Respir. Crit. Care Med. 1995 (22)	Randomized double-blinded, placebo-controlled study	Six ILD patients (Three IPF).	Nebulized morphine 2.5 mg and 5.0 mg.	2.5 or 5 mg	Modified Borg scale for dyspnea at end exercise after cycle ergometer test (HR, ECG, sat-O_2_, inspiratory and expiratory volumes, expired O_2_, and CO_2_).	No significant differences in any of the measured variables including dyspnea.
Masood et al., Thorax 1995 (28)	Randomized, double-blinded, placebo-controlled, crossover study	12 COPD patients.	Combinations of nebulization and i.v. treatment in five regimens: 1) placebo, 2) nebulized morphine 10 mg, 3) nebulized morphine 25 mg, 4) i.v. morphine 1.0 mg, and 5) i.v. morphine 2.5 mg. With 2 + 3 i.v. placebo and with 4 + 5 nebulized placebo was given.	10 or 25 mg, 3.0 or 7.5 mg	100 mm VAS scale for dyspnea at rest and during cycle ergometer test (oxygen consumption (VO_2_), carbon dioxide production (VCO_2_), FEV1, VC, PEFR, and sat-O_2_. HR, RR, BP, exercise endurance, symptom limitation, and plasma morphine level.)	No significant change in dyspnea. No change in exercise endurance or ventilation.
Leung et al., Thorax 1996 (25)	Randomized, double-blinded, placebo-controlled, crossover study	Nine COPD patients and one ILD patient.	Nebulized morphine 5 mg (1 g/ml).	5 mg	Modified Borg score for dyspnea every minute during exercise on cycle ergometer (sat-O_2_, RR, MV, and ECG, dose of nebulized morphine).	No significant difference in dyspnea or power output.
Jankelson et al., Eur. Respir. J. 1997 (24)	Randomized double-blinded, placebo-controlled, crossover study	16 COPD patients.	Nebulized morphine sulfate 20 or 40 mg.	20 or 40 mg	Modified Borg score for dyspnea at 6MWT (sat − O_2_, HR, walked distance. Spirometry (FEV1 and FVC) and measure of blood gases at baseline. Plasma level of morphine).	No significant change in dyspnea or other parameters.
Noseda et al., Eur. Respir. J. 1997 (20)	Randomized, double-blinded, placebo-controlled crossover study	17 patients (11 COPD, 1 cancer, 1 IPF, 1 heart disease).	Nebulized morphine given in four settings: morphine 10 mg×2 (with and without O_2_), morphine 20 mg with oxygen or saline with oxygen. Oxygen was given 3 out of 4 days.	10 or 20 mg	Bipolar (−100%, +100%) VAS scale for dyspnea at rest (sat-O_2_, RR).	No significant difference in dyspnea. No difference in response to 10 mg morphine given with/without oxygen.
Jensen et al., J. Pain Symp. Man. 2012 (29)	Single center, randomized, double-blind, placebo-controlled crossover study	12 COPD patients.	Nebulized fentanyl citrate 50 mcg (single dosage).	5 mg	Dyspnea intensity at isotime during exercise and EET (PFT, cardiorespiratory, and breathing pattern parameters measured at rest, isotime, and at end of exercise).	No significant change in dyspnea intensity. Significantly increased exercise endurance time.

a The studies by Eiser and Light each consisted of two different studies and they were therefore evaluated separately.

COPD: chronic obstructive lung disease; t.i.d.: three times a day; VAS: visual analog score; FEV1: forced expiratory volume in 1 sec; FVC: forced vital capacity; PEFR: peak expiratory flow rate; 6MWT: 6-min walk test; MP: promethazine; MPC: prochlorperazine; HR: heart rate; RR: respiratory rate; VO_2_max: maximal oxygen consumption; VE: minute ventilation; VCO_2_max: maximal CO_2_ production; VT: tidal volume; sat-O_2_: arterial oxygen saturation; q.d.: once daily; BP: blood pressure; ILD: interstitial lung disease; IPF: idiopathic pulmonary fibrosis; RF: respiratory frequency; ECG: electrocardiogram; i.v.; intravenous; PFT: pulmonary function test; SE: standard error.

## Results

### Study design

Twelve of the fourteen studies selected for review were randomized, placebo-controlled crossover studies (14, 17). The patient populations were heterogeneous and different routes of morphine administration were used (Table 1).

In total, the 14 studies included 311 patients (*n*=32, 10% IPF/ILD) who were all opioid-naïve at inclusion and for minimum 1 week prior to inclusion. The sample sizes were small, with 6–83 patients in each study. Dyspnea was the main symptom investigated in all 14 studies and was used as the inclusion criteria in 10 studies. Dyspnea was measured as an outcome in all but one study (18).

### Test conditions

Five studies investigated patients with dyspnea at rest (14, 17, 19–21), six studies included data during exercise alone (18, 22–25), and three studied the effects of opioids on dyspnea both at rest and during exertion (26–29). Different exercise tests were used, such as the 6-min walk test (6MWT), cycle ergometer test, and treadmill walk test (Table 1).

### Dyspnea score

Different scores were used in order to assess the level of dyspnea and the potential effect of morphine. The most commonly used was the visual analog scale (VAS), used in seven studies. This score is sensitive (30) and reproducible (31). A modified Borg score and a Likert score were also used. In the study by Young, no score was used (18).

The minimal clinical important difference (MCID) was described by Miriam Johnson et al. (32). They set up 9 mm as a significant change in a 100-mm dyspnea VAS scale based on four clinical trials. Currow (17) referred to a study by Ries suggesting that the MCID is 1 unit for the Borg scale and 10–20 units for the VAS (33). Currow also statistically explored the clinically important difference and found it to be 50% of the standard deviation of the baseline observation, giving an MCID of 9.9 mm.

The frequency of measurements varied between studies. Long-term observations were made by Currow (17) (3 months), Johnson (26) (2 weeks), Poole (19) (6 weeks), and Eiser (27) (2 weeks). Measurements were most often obtained twice daily, but by Johnson only in the evening and by Eiser (27) only reported as ‘by daily diary cards’. In studies involving an exercise test, dyspnea was most commonly measured at fixed 1-min intervals (22, 24–26, 28). Light (23) measured dyspnea during the last 30 sec at each power output and Eiser (27) only immediately after exercise. Young (18) only investigated if dyspnea was the limiting symptom, without using any score.

### Route of opioid administration, dose, and compliance

Different types of opioids were used. Equivalent morphine doses are shown in Table 1. Six studies investigated morphine administered by inhalation (18, 20, 22, 24, 25, 28) and six studies investigated orally administered morphine (17, 19, 21, 23, 26, 27). A single study used subcutaneous injections of morphine followed by long-term oral morphine (14), and one study used inhaled fentanyl (29). The plasma levels of morphine after administration were measured by Masood (28), Jankelson (24), and Eiser (27). Compliance was assessed in two studies (19, 27). Poole counted the number of tablets left after the intervention period and found that more than 90% of tablets were taken by all participants.

### Effect of opioids

A beneficial effect of morphine on dyspnea was observed in four studies (14, 17, 21, 26). The most prominent effect was reported by Allen (14), who found a 50-mm decrease in a dyspnea VAS scale, 15 and 30 min after injection of 2.5/5 mg morphine (5 mg for patients >50 kg) subcutaneously. In the other studies, the difference in VAS ranged from 9.5 to 50 mm. An effect on exercise tolerance was reported in two studies (18, 26). One study found a beneficial effect on both dyspnea and respiratory parameters with combined morphine and promethazine, but not with morphine alone (23). Morphine had no effect on dyspnea in seven other studies (19, 20, 22, 24, 25, 27, 28). The study with inhaled fentanyl showed a positive effect on exercise capacity, but not on dyspnea (29).

### Adverse effects

None of the studies reported any serious side effects. In order to study the risks of respiratory depression, nearly all studies investigated the effect on oxygen saturation or other parameters related to respiratory function (Table 1). None of the studies found any signs of respiratory depression.

Constipation was the reason for cessation of morphine and withdrawal among eight patients in one study (17). It was also found to be a common side effect based on complaints from 37 participants in another study (19). Nausea, vomiting, drowsiness, and sedation were also reported by patients, although less frequently. Withdrawals were observed in six studies. In a single study (17), the number needed to harm was found to be 4.6 and the number needed to treat 1.6. No studies reported on hospitalization caused by adverse effects.

## Discussion

The main finding of the present study was that only very few randomized, placebo-controlled studies had investigated the effect of opioids in IPF and that systemic administration seemed to have a beneficial effect on the perception of dyspnea, compared with inhaled morphine. However, due to the paucity of studies in IPF, studies including patients with COPD were included.

Different end points and variable routes of administration and doses of opioids were used in the studies included. A beneficial effect of morphine on dyspnea was observed in four studies (14, 17, 21, 26) and an effect on exercise tolerance in two studies (18, 26). One study found a beneficial effect of morphine combined with promethazine (23). All positive studies used systemic administration of morphine. Lack of effect was reported in seven studies (19, 20, 22, 24, 25, 27, 28), five of which used inhaled morphine (20, 22, 24, 25, 28). Constipation was reported as an adverse effect to morphine treatment, but respiratory depression, the most feared side effect, was not observed in any of the studies.

Overall, the studies were small but well designed. Only 2 of 14 studies were not placebo-controlled. However, both of these studies (14, 17) showed a positive effect of morphine; due to the lack of a control group, these results should be interpreted with caution.

Studies including patients with COPD were included as studies in IPF were few. Differences between the two groups were described in a study (16) measuring arterial oxygen saturation (sat-O_2_) and dyspnea score during 6MWT. Desaturation was found to be more severe in IPF, although dyspnea was milder compared to patients with COPD at the same walking distance. However, both groups complained of dyspnea as the main symptom and might benefit equally from the potential effect of opioids as found during subgroup analysis by Jennings et al. (15). However, some COPD phenotypes resemble IPF more than others. The subgroup of IPF patients with combined pulmonary emphysema and fibrosis (CPFE) (34) forms a specific phenotype and shares many common traits with other COPD phenotypes. CPFE is characterized by more severe dyspnea and more frequent prevalence of pulmonary hypertension. It can be speculated that this phenotype and COPD phenotypes such as those with very severe emphysema will benefit more from opioid treatment than those with a chronic bronchitis phenotype. Whether differences in COPD phenotypes contribute to the diversity of the results of the different studies is hard to interpret, and therefore studies in IPF patients are still necessary.

The difference in route of administration and dose of opioids seemed to be the most obvious difference between positive and negative studies. Studies without effect of opioids most commonly used the intrapulmonary route of administration by nebulization. The nebulized administration form was chosen to target the opioid receptors in the respiratory tract and thus to benefit from the local effect and avoid adverse systemic effects. Yet no studies using nebulization detected any effect of morphine on dyspnea. Therefore, it seems that central mechanisms for dyspnea are more important in IPF and COPD than local intrabronchial mechanisms. However, the intrapulmonary dose was measured by weighing the nebulizer before and after inhalation in accordance with Harris-Eze (22) and Leung (25), whereas plasma levels were measured by Masood (28), Jankelson (24), and Eiser (27). The relevance of measuring a plasma level was seen in the study by Eiser (27) as 14 out of 27 blood samples showed no detectable morphine. One explanation for the low plasma levels is poor compliance and Eiser did control for this by measuring the volume of medicine/placebo left at the end of each study period. Only two patients had taken all 56 prescribed doses. Detectable levels ranged between 1.8 and 9.2 ng/ml according to Eiser. The conclusion by Jankelson was that on completion of nebulizing 40 mg of morphine, the plasma levels resembled those achieved 15 min after intravenous administration of 1–2.5 mg of morphine. Thus, Jankelson considered plasma levels as capable of producing a clinical effect. In the study by Masood (28), both nebulized and intravenously administered morphine were used and plasma levels were significantly higher in patients receiving intravenous morphine, although the intravenous dose was 10 times lower than the nebulized dose. The average plasma level before exercise after 2.5 mg of intravenous morphine was 25.0 ng/ml (standard error (SE) 3.3). The average plasma level before exercise after nebulization of 10 mg of morphine was 2.5 ng/ml (SE 0.3). However, this study did not find any effect of intravenous morphine. Another explanation for the varying results may be the use of a standardized dosage of morphine versus an individualized dosage based on patient weight, thereby possibly risking either over- or under-treatment. Moreover, the dose of morphine was interestingly similar within three of the studies showing a beneficial effect of morphine. Allen (14) used diamorphine equivalent to 10 or 20 mg of oral morphine adjusted by weight. Currow (17) and Abernethy (21) used 10–30 mg titrated to effect but without side effects. These three studies all reported an effect on dyspnea at rest. A lower dose of morphine was used by Young (18) and Johnson (26) (Table 1). Thus, systemic administration and an individually titrated dose between 10 and 30 mg seem to be associated with a beneficial effect on dyspnea. The lack of effect seen in most studies using inhalation may be explained by sub-therapeutic doses.

The conditions under which dyspnea and exercise tolerance were measured is another important issue. Some patients were tested at rest in bed while others were tested at exertion to Emax on a cycle ergometer. Each constellation is relevant for understanding the effect of morphine. However, the most strenuous exercises are likely to occur only during testing and not during daily living. The studies claiming improved exercise tolerance after morphine treatment used cycle ergometer and treadmill tests. The four studies showing an effect of morphine on dyspnea mainly measured dyspnea at rest – in a hospital bed (14) or daily at home (17, 21, 26). Johnson (26) measured both dyspnea with the patient at home and during a treadmill test and detected a benefit of morphine on dyspnea in both situations. Thus, morphine seems to be equally effective for dyspnea both at rest and during exercise. The level of dyspnea at baseline may influence the effect of morphine. Allen reported the largest reduction in dyspnea and explained this finding with the fact that their participants were terminally ill and likely to experience a proportionally greater reduction of dyspnea compared to less dyspneic patients. Moreover, the setting, with measurement of immediate relief as opposed to self-reported dyspnea scores over several days and weeks, may have influenced the results.

The concern of respiratory depression was addressed by most studies. Currow stated that the concern is based on ‘the acute toxicity witnessed when frail or infirm patients who were opioid-naïve were administered opioids in the emergency room or postoperatively’ (17), which could not and should not be compared with the administration of low-dose morphine. Two studies (21, 17) did not measure patient ventilation. Saturation was measured in all studies except four (17, 18, 23, 26), and no significant change in ventilation was detected in any of the studies. Other side effects, such as nausea and constipation, occurred but were treated with laxatives and anti-emetics (15). Additionally, nausea and constipation might be tolerable to some extent considering the possible improvement in the perception of dyspnea (21).

In conclusion, it remains unclear whether morphine is beneficial for the treatment of dyspnea in patients with IPF. The results suggest a beneficial effect of morphine in sufficient doses on patient-important outcomes. Some studies suggest a positive effect if sufficient systemic doses of morphine are used, while no effect was detected in others; most studies used lower morphine doses and an intrapulmonary route of administration. Large-scale randomized placebo-controlled trials in patients with IPF are very much needed to clarify whether morphine is effective in the treatment of dyspnea.
